# Managing threatened ungulates in logged-primary forest mosaics in Malaysia

**DOI:** 10.1371/journal.pone.0243932

**Published:** 2020-12-14

**Authors:** Mark Rayan D., Matthew Linkie

**Affiliations:** 1 World Wide Fund for nature (WWF) Malaysia, Petaling Jaya, Selangor, Malaysia; 2 Durrell Institute of Conservation and Ecology (DICE), University of Kent, Canterbury, United Kingdom; 3 Wildlife Conservation Society (WCS), Bogor, Indonesia; Bowling Green State University, UNITED STATES

## Abstract

Across the tropics, large-bodied mammals have been affected by selective logging in ways that vary with levels of timber extraction, collateral damage, species-specific traits and secondary effects of hunting, as facilitated by improved access through logging roads. In Peninsular Malaysia, 3.0 million hectares or 61 percent of its Permanent Reserved Forests is officially assigned for commercial selective logging. Understanding how wildlife adapts and uses logged forest is critical for its management and, for threatened species, their conservation. In this study, we quantify the population status of four tropical ungulate species in a large selectively logged forest reserve and an adjacent primary forest protected area. We then conduct finer scale analyses to identify the species-specific factors that determine their occurrence. A combined indirect sign-camera trapping approach with a large sampling effort (2,665 km and 27,780 trap nights surveyed) covering a wide area (560 km^2^) generated species-specific detection probabilities and site occupancies. Populations of wild boar were widespread across both logged and primary forests, whereas sambar and muntjac occupancy was lower in logged forest (48.4% and 19.2% respectively), with gaur showing no significant difference. Subsequent modelling revealed the importance of conserving lower elevation habitat in both habitat types, particularly <1,000 m asl, for which occupancies of sambar, muntjac and gaur were typically higher. This finding is important because 75 percent (~13,400 km^2^) of Peninsular Malaysia’s Main Range Forest (Banjaran Titiwangsa) is under 1,000 m asl and therefore at risk of being converted to industrial timber plantations, which calls for renewed thinking around forest management planning.

## Introduction

The worldwide trade in tropical timber from managed natural forests is estimated at US$11.2 billion a year [1]. The majority of these logs are extracted from Asian forests, but timber production in many countries in this region peaked several decades ago due to over-harvesting and forest conversion [2]. The effects of this logging on tropical wildlife varies amongst taxa [3], even when the over-riding effects of hunting are controlled, and are primarily explained by logging intensity, logging techniques and their associated collateral damage, as well as wildlife species plasticity [4–7].

The impacts of logging vary among mammalian species. For example, some species experience negative impacts from logging including the loss of fruiting trees on mouse deer (*Tragulus* spp.) and yellow muntjac (*Muntiacus atherodes*) [8] or the disruption of canopy integrity on brachiating gibbons [9]. In contrast, the disruption of the forest canopy through selective logging increases sunlight penetration into the understory and stimulates herbaceous plant growth that can offer benefits to browsing and grazing ungulates such as sambar (*Rusa unicolor*) and gaur (*Bos gaurus*) [10]. Such disruption often comes as a cost of new roads being developed, which in turn facilitate hunter access to previously remote and therefore safer forest refuges for large-bodied ungulates and trophy species, such as large carnivores [11].

In Peninsular Malaysia, about three million hectares or 61 percent of its Permanent Reserved Forest is primarily assigned for commercial selective logging through its designation as Production Forest. The Forestry Department of Malaysia requires the use of reduced-impact logging practices that include pre-harvest planning, which includes identification and demarcation of sensitive areas such as road and stream buffer zones. It also includes minimising skidding distances, directional felling and post-harvest site closure [12]. Compared to conventional logging, reduced-impact logging reportedly has fewer impacts on wildlife because it reduces the collateral damage associated with timber felling and allows for more rapid post-logging forest recovery [3,13]. However, how reduced-impact logging is implemented in practice remains an important area of research in Malaysia and a more recent threat has been clearance within selectively logged forests for timber latex clone rubber plantations [14]. Permanent Reserved Forests are now under threat by expansions of timber plantations which are targeted to expand from 1,626 ha in 2006 to 375,000 ha by 2020 [14], of which 120,000 ha of forest plantations have thus far been established [15]. A new set of standards called the Malaysian Criteria and Indicators for Sustainable Forest Management established in 2020, allows 5% of the certified forests in a state to be converted to plantations. Nevertheless, the threat of poorly managed logged-over forests being classified as degraded forest and then converted to other land uses, remains an imminent threat to preserving natural forest within Peninsular Malaysia.

Malaysia is a biodiversity-rich country and given its prominence, Permanent Reserved Forest should play an important role for much of Peninsular Malaysia’s threatened wildlife. For example, this land use type represents 85 percent of Malaysia’s presumed tiger habitat, with co-occurring populations of leopard (*Panthera pardus*) and dhole (*Cuon alpinus*) [16]. These forests support a variety of ungulate species that constitute the prey of these threatened carnivores [17], such as gaur and sambar, that are both categorized as Vulnerable on the IUCN Red List and also red muntjac (*Muntiacus muntjac*) and wild boar (*Sus scrofa*), listed as Least Concern. Yet, the population status and ecology of these ungulates under different forest management regimes is poorly understood but greatly needed for better management.

In this study we aim to conduct the first occupancy-based assessments of a variety of threatened and non-threatened ungulate species living in a Permanent Reserved Forest and an adjacent primary forest protected area in the northern Peninsular Malaysia state of Perak. We use a combined dataset from indirect sign surveys and camera traps, to compare the efficiency of these two methods in sampling difficult-to-detect species. In addition, we used results from an interview survey conducted from May-September 2010 with 190 Orang Asli respondents from both study areas (92 in the logged-over Permanent Reserved Forest and 98 in the primary forest protected area) to supplement our ecological findings [18].

## Materials and methods

### Study area

**Fig 1:**
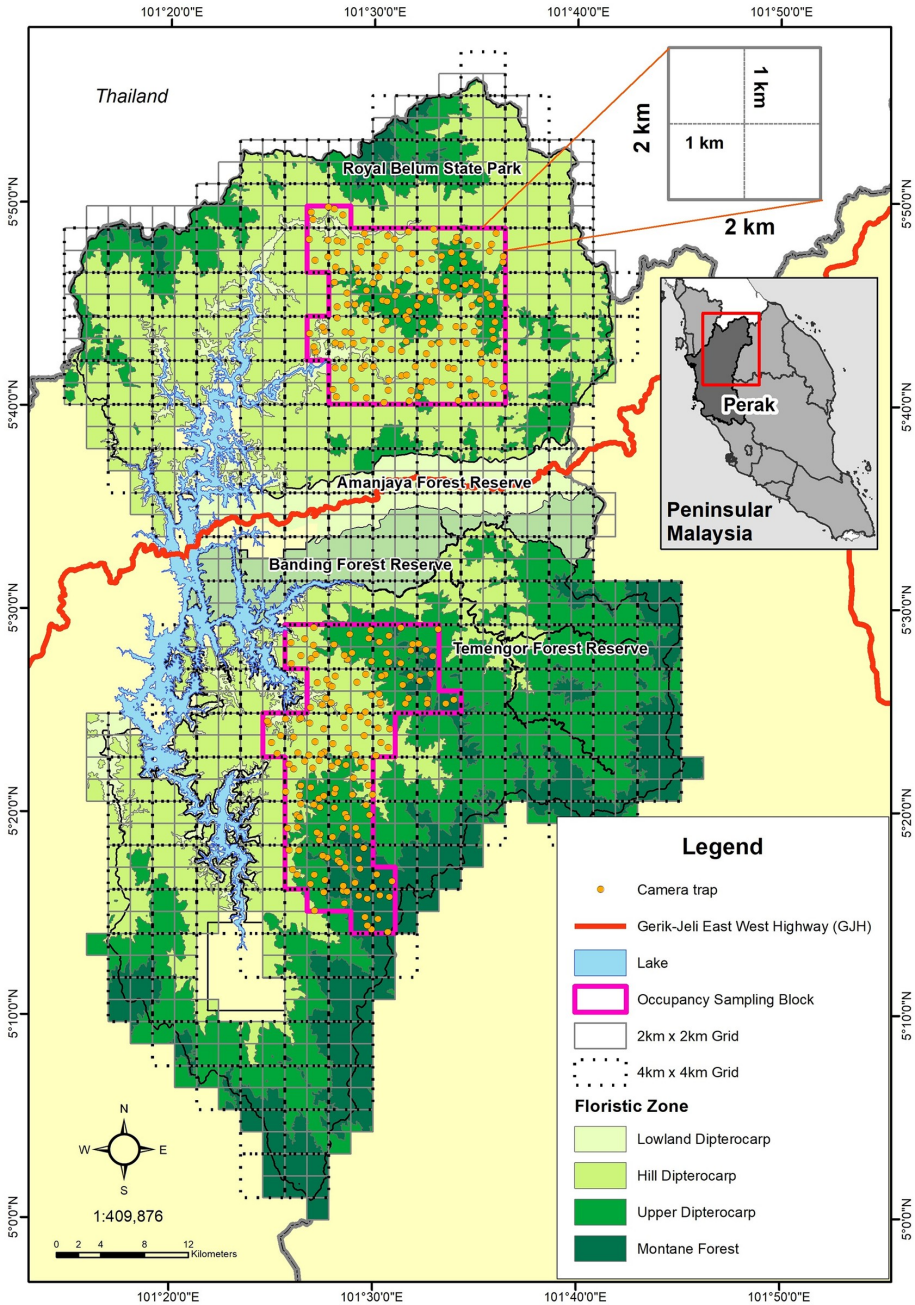
Location of the study areas of Temengor Forest Reserve and Royal Belum State Park, occupancy sampling block and camera traps.

The two study areas the Royal Belum State Park (RBSP) and Temengor Forest Reserve (TFR) are both located in the state of Perak in Peninsular Malaysia (101°15ʹ0ʺ- 101°46ʹ0ʺE and 5°55ʹ0ʺN—5°0ʹ0ʺN; Fig 1). The 1,175 km^2^ RBSP was officially gazetted in 2007 as a strictly protected area with only non-exploitive commercial activities permitted, such as limited tourism. The altitudinal range of 260 m to 1,533 m above sea level (asl) supports lowland dipterocarp (5.6%), hill dipterocarp (71.5%), upper dipterocarp (20.9%) and montane (2.0%) forest types. Being located by the southern Thai border, which has a long-running history of insurgency, there has been a Malaysian army presence in RBSP since the late 1940s. In contrast, we compared the unlogged forest of RSBP with the 1,489 km^2^ TFR, which was officially gazetted in 1991 as a Permanent Reserved Forest and has been undergoing selective logging since the 1970s. The altitudinal range of 260 m to 2,160 m asl supports lowland dipterocarp (4.2%), hill dipterocarp (34.4%), upper dipterocarp (41.7%) and montane (19.7%) forest types. The TFR has no specific wildlife management plan or prescriptions and an estimated 5,000 indigenous people reside within 24 settlements in and around the area, whereas RBSP has an estimated 740 indigenous people (Department of *Orang Asli* Affairs unpublished data).

### Field survey design

Occupancy surveys of gaur, sambar, muntjac and wild boar i.e., focal tiger prey species, were conducted from August 2009-May 2010 in TFR and August 2010-April 2011 in RBSP using a combination of camera trapping and indirect sign surveys. The surveys were conducted within the similar period between both years to minimise climatic variation on the observations, as generally apart from the increased rainfall affected by the north-east and south-west monsoon, the climate is humid throughout the year.

A 4 km^2^ grid system was overlaid on the two study areas and a stratified systematic sampling approach then used, based on the main floristic zones that gave proportional coverage of the main habitat types for each study area. This was achieved by the configuration of 70 x 4 km^2^ grid cells (occupancy block) in each study area in which both sign and camera trapping surveys were conducted (Fig 1).

Species detection through three indirect sign surveys over nine months was conducted by six teams, each of two personnel (a field biologist and an indigenous guide). Each team was rotated in sequence to survey each sampling unit once, thereby minimising observer bias between temporal replicates and to avoid introducing heterogeneity into detection probabilities. Each team was required to survey a minimum of 1 km per 1 km^2^ sub-grid cell. Within each 4 km^2^ grid cell, the two-person teams intensively searched areas thought to have the highest likelihood of focal species sign (i.e. tracks < 1 month old), such as forest trails, ridges, sand beds, river banks, saltlicks and logging roads. Signs of focal species and weather condition (rain or no rain within a 24-hour period) were recorded within each sampling unit.

Camera trapping was first conducted for approximately nine months in TFR and subsequently another nine months in RBSP, with a sampling area of 280 km^2^ in each study area. A 140 single camera traps were placed at locations considered as most likely to record prey (i.e. trails such as on ridges or old logging roads) within the 4 km^2^ km grid system. Single traps for prey occupancy were moved once, during the third or fourth month, within the 4 km^2^ grid cell to increase spatial trapping coverage. Custom-built passive-sensor digital camera trap units (Sony P41 digital cameras housed in a waterproof pelican case), programmed to take photographs at ten second intervals between detections, were used. Camera traps were set to be operational for 24 hours each day with no break in monitoring, except during events of malfunction or damage. All camera traps were mounted on trees at a height of approximately 50 cm from the ground, depending on the slope of the trail. Camera traps were checked every 2–3 months to retrieve data and replace batteries.

As the home range sizes of gaur, sambar, wild boar and muntjac in Malaysia are not known, a scaling model that expresses the relationship between body mass and home range size was used to estimate these sizes [19]. This then determined the appropriate sample unit size for each species.

The average body mass (*M*) in kilograms was multiplied by the observed scaling relationship per individual area used (*m*), taken as 2.06 for herbivores. This value was then raised to the power of a calculated scaling exponent (*h*), which is 1.02 for herbivores, resulting in the species-specific home range size estimates. The values for *m* and *h* were obtained from Jetz et al. (2004) [19] and *M* from Kawanishi and Sunquist (2004) [17], with the exception that *M* for wild boar that was obtained from Ickes et al. (2001) [20]. The average body weights used for gaur (450 kg), sambar (134 kg), wild boar (62 kg) and muntjac (21 kg) corresponded to respective home range sizes of 10.5 km^2^, 3.0 km^2^, 1.4 km^2^ and 0.4 km^2^. Sampling units of 16, 4, 4 and 1 km^2^ were used to define a sampling unit that was larger than the approximate home range values for gaur, sambar, wild boar and muntjac, respectively. This definition of a larger arbitrary sampling unit size is therefore an additional criterion to ensure that occupancy and not habitat use was being measured. Four 4 km^2^ grid cells were combined to create a sampling unit for gaur (16 km^2^) yielding 22 cells in TFR and 20 in RBSP. The 4 km^2^ grid cell was divided into four sampling units for muntjac (1 km^2^) yielding 280 cells per study area, whereas for sambar and wild boar the original number of 4 km^2^ sites per study area (n = 70) were retained.

### Single species occupancy analysis

Occupancy (*ψ*), defined as the probability of occurrence within a sampling unit, was estimated from a combination of camera trap and indirect sign survey data within a likelihood-based sampling approach. Individual prey species detection histories (H) were constructed for each site (sampling unit). This included 10 four-week sampling occasions for camera trapping and three temporal occasions consisting of one independent sign survey each, similar to a multiple method approach used by Long et al. (2011) [21]. For each site and for each occasion, ‘1’ indicated the detection (photograph or sign; tracks < 1 month old) of a species, ‘0’ indicated the non-detection of a species and ‘-’ indicated that either there was no functioning camera trap in place or that no sign surveys were carried out in that particular occasion. In cases where there was more than one camera trap per sampling unit (such as 4 km^2^ grid cells being merged for gaur sampling), detections were aggregated to produce a single detection history for a particular sampling unit based on the corresponding calendar dates. Though this approach will inevitably cause the effort applied in each sampling occasion to be uneven, this is dealt with by incorporating a trap effort covariate to account for possible variation in detection probability. Species detection histories from both indirect sign surveys and camera trapping were used to produce probabilities in which detection probability (*p)* was varied in the design matrix to be conditional upon each method.

Data from the sampling units for the two study areas were combined. The two main disturbances to the habitat within each study area are logging (in TFR) and permanent human presence (indigenous settlements in RBSP and TFR, and logging camps in TFR). In terms of the habitat topography, the altitudinal range in TFR is greater than in RBSP. The proximity of the road bisecting TFR and RBSP in relation to the sampling blocks was deemed as being too far away (>7 km) to directly influence the occupancy of prey species within the study areas [22] and was not included as a covariate. The spatial dataset consisted of four continuous covariates; (i) distance to settlement, (ii) mean elevation, (iii) logging intensity index, and (iv) mean Normalized Difference Vegetation Index (NDVI), was derived for the 1, 4 and 16 km^2^ sampling units.

To test for collinearity between the continuous covariates, a Spearman’s rank correlation was performed. Covariates were considered to be highly correlated if their corresponding coefficients were >0.6. This test identified significant (*P* < 0.05) non-independence between most of the continuous variables across the three sampling unit scales (1, 4 and 16 km^2^) but the correlation coefficients (r_s_) were all <0.6, except for two variables at two scales; logging intensity index (4 km^2^, rs = -0.67) and mean NDVI (16 km^2^, r_s_ = -0.75). Although NDVI can be used as a proxy to describe the structural vegetation disturbance caused by logging, a logging intensity index was used instead because it measures disturbances caused by logging on a temporal scale (years logged since 2010) and a spatial scale (size of area logged according to year).

Obtaining detailed information on the volume or number of logged trees was not possible. Instead, we used information provided by the State Forestry Department of Perak to calculate a logging intensity index for each sampling unit, whereby the size of the logged area was multiplied by the reciprocal number of years logged since 2010. For example, if a sampling unit contained two patches of forest that were respectively logged in 2010 and 2009 and covered 0.2 and 0.1 km^2^, the resulting index would be calculated as ‘((1/1 x 0.2) + (1/2 x 0.1)) = 0.25’. For RBSP, the logging intensity index was zero for all sampling units because it represents unlogged forest. At the 1 km^2^ scale, the correlation coefficient was close to 0.6 (r_s_ = -0.58) between logging intensity and mean NDVI. Hence, in order to standardize the use of covariates across all prey species at all scales, mean NDVI was not used in the analysis.

To account for uneven detection probability in all species except gaur, five covariates were tested: (i) survey effort per grid cell (distance walked adjusted for varying topography by overlaying two-dimensional Global Positioning System tracklogs onto a three-dimensional digital elevation model); (ii) number of trap-nights a camera trap or multiple camera traps (sum of trap-nights for cases where a camera trap in the same sampling unit or sum of trap-nights for all traps within a sampling unit where grid cells were combined for gaur) were operational within a site; (iii) a binary variable for whether it had rained (1) or not (0) within a sampling unit 24-hours prior to the survey (which may influence indirect sign detection); (iv) categorical variables (1–5) assigned for each observer (excluding the reference observer) to account for potential bias between observers; and (v) a binary study area covariate. As four 4 km^2^ grid cells were combined to create a sampling unit for gaur, it was not possible to incorporate detection probability covariates for rain and observer bias due to the survey design, where for each survey each observer surveyed different 4 km^2^ grid cells; a 16 km^2^ grid cell was also typically surveyed over 3–4 days with varying levels of rainfall. Therefore, only survey effort (distance walked and sum of trap-nights) and a study area covariate were used to account for gaur detection probability. Continuous covariates were transformed into standardized z-scores prior to analysis.

The combination of covariates that best explained occupancy for each prey species and detection probabilities were investigated using PRESENCE v4.0 software [23] under the single-species, single-season framework. The single-season multi-method framework was not used because there was no reason to assume a lack of independence between indirect sign survey and camera trap survey methods, as required [21]. A two-step approach was used to model parameters of interest. First, detection probability (*p*) was modelled where the parameter was either assumed constant or allowed to vary with individual or additively combined covariates. For each model, a global model for the probability of occupancy (*ψ*) was maintained. Subsequently, the influence of covariates on occupancy was modeled where the parameter was either assumed constant or allowed to vary with individual or additively combined covariates, whilst maintaining the top ranked model for detection probability as derived from the first step.

Candidate models were ranked using the small-sample correction to Akaike’s information criterion (AICc) [24] by changing the effective sample size (defined as the number of sampling sites). Model fit was evaluated by comparing the observed Pearson chi-square statistic from the global model with chi-square statistics from 10,000 simulated parametric bootstrap datasets [25]. Poor model fit (i.e. $\hat{c}$ >1.0) was accounted for by estimating an over-dispersion factor ($\hat{c}$) with inflated corresponding standard errors ($\sqrt{\hat{c}}$). In cases of poor model fit, a quasi-likelihood over-dispersion parameter (QAICc) for model selection was used [24].

Covariates that were likely to affect detection probability and occupancy probabilities were identified based on the covariates that were contained in the top ranked model and relative summed Akaike weights (Ʃ*w*_*i*_) of models that contained a particular covariate; where Ʃ*w*_*i*_ > 0.50 were considered indicative of a strong habitat use response to a covariate [26]. In addition, parameter estimate coefficients from the top ranked model which form a logistic regression equation were used to plot the predicted occupancy for each species. To obtain overall occupancy estimates for each species according to each study area, if no model received a *w*_*i*_ >90%, occupancy estimates were model averaged and corresponding standard errors calculated [24] based on an approach similarly used by other studies [27,28].

Differences in the study area model averaged occupancy estimates ($\hat{\psi }$) for each species were assessed using a Wald test, whereby Z values larger than 1.96 (critical value at α = 0.05) were considered significant. To assess the degree of spatial autocorrelation in response variables not accounted for by predictor variables affecting model averaged occupancy estimates, Moran’s *I* statistic [29] was calculated using the Crime-Stat v1.1 software (N Levine and Associates, Annadale, VA) for model-averaged residuals [30].

### Implications for future survey designs

The occupancy analysis was conducted with data combined from indirect sign surveys and camera trapping, which were expected to increase the probability of species detection. To compare the effectiveness of the two survey methods to detect the focal prey species, a single dataset for both study areas combined was used to generate comparative naive detection probabilities. The detection efficiency was assessed based on the naive estimates; higher naive occupancy estimates indicate a higher number of unique detections. Next, the probability of a false absence (1- *p*)^No. surveys(K)^ for each prey species according to each method was determined by individually analysing each method, where estimates of *p* were derived from constant models, *ψ*(.)*p*(.). These estimates were compared between methods for each species, whereby probabilities greater than 0.15 were considered as unacceptable to make an inference on occupancy patterns for a population [31]. Even though multiple detections of the same individuals from a species may be recorded within a sampling unit, it is important to note that occupancy and detection probabilities are measured at the species level and not at the individual animal level [31].

Based on the comparisons of naive occupancies and false absences, the survey method that performed better in terms of detection efficiency was further evaluated to provide recommendations in terms of the number of sites that would be required to be surveyed for future sampling. Recommendations developed by MacKenzie and Royle (2005) [32] were used to determine the optimal number of sampling occasions (*K*), to obtain occupancy estimates with a specified improved precision (i.e. Standard Error (SE); Variance, $Var(\hat{\psi })$ = 0.05). For this analysis, assumed occupancies and detection probabilities were based on the constant model for each species in which these estimates were used to compute the number of sites (s) based on the occupancy variance equation prescribed by MacKenzie et al. (2006) [33]. All necessary approvals from the Perak State Parks Corporation, the Department of Wildlife and National Parks and the Forestry Department of Perak were acquired prior to conducting this study. Due to the non-invasive nature of this observational field study, no special permits or ethical approval was required for the described study, which complied with all relevant regulations.

## Results

### Species detection probability

The two study areas had high and similar levels of sampling effort: TFR (1,410 km walked over three repeat surveys and 13,808 camera trap night); and RBSP (1,255 km walked over three repeat surveys and 13,972 trap nights). Based on these data, detections and naive occupancies of all species were found to be higher in RBSP than in TFR (S1 Table). The high naive occupancies for wild boar in TFR (0.96) and RBSP (1.00; S1 Table), meant that only the *ψ*(.)*p*(zone) model was used to subsequently investigate differences in detection probabilities between methods and study areas. The estimated wild boar detection probability from sign surveys and camera trapping showed variation in detection probability between study sites and methods in which detection probability was significantly higher in RBSP ($\hat{p}$± SE; 95% Confidence Intervals (CI): 0.52±0.02; 0.48–0.57) compared to TFR (0.39±0.02; 0.35–0.43) for camera trap surveys but was not significantly different for sign surveys in RBSP (0.83±0.03; 0.77–0.87) and TFR (0.77±0.03; 0.70–0.82).

The constant *p*(.) model performed poorly for sambar (ΔAICc = 145.3), muntjac (ΔAICc = 296.7), and was not highly ranked for gaur (ΔAICc = 3.3). The percentage of relative summed model weights for covariates influencing detection probability showed a high level of support for covariates contained within the top ranked model for all species, in which gaur detection probabilities were highly affected by sign survey effort (distance walked), for sambar it was zone, rain and camera trapping effort (trap-nights), and for muntjac it was zone, observer sign detectability variation, camera trapping effort and sign survey effort (S2 Table). Detection probability was higher in RBSP compared to TFR (for sambar and muntjac), for Observers 1, 3 and 4 and the reference observer compared to Observer 2 and 5 (for muntjac), when it did not rain within a 24-hour period (for sambar), presumably because tracks were washed away by rain, when camera trapping effort was higher (for sambar and muntjac) and when longer distances were walked within a sampling unit (for gaur and muntjac).

### Species occupancy

**Fig 2:**
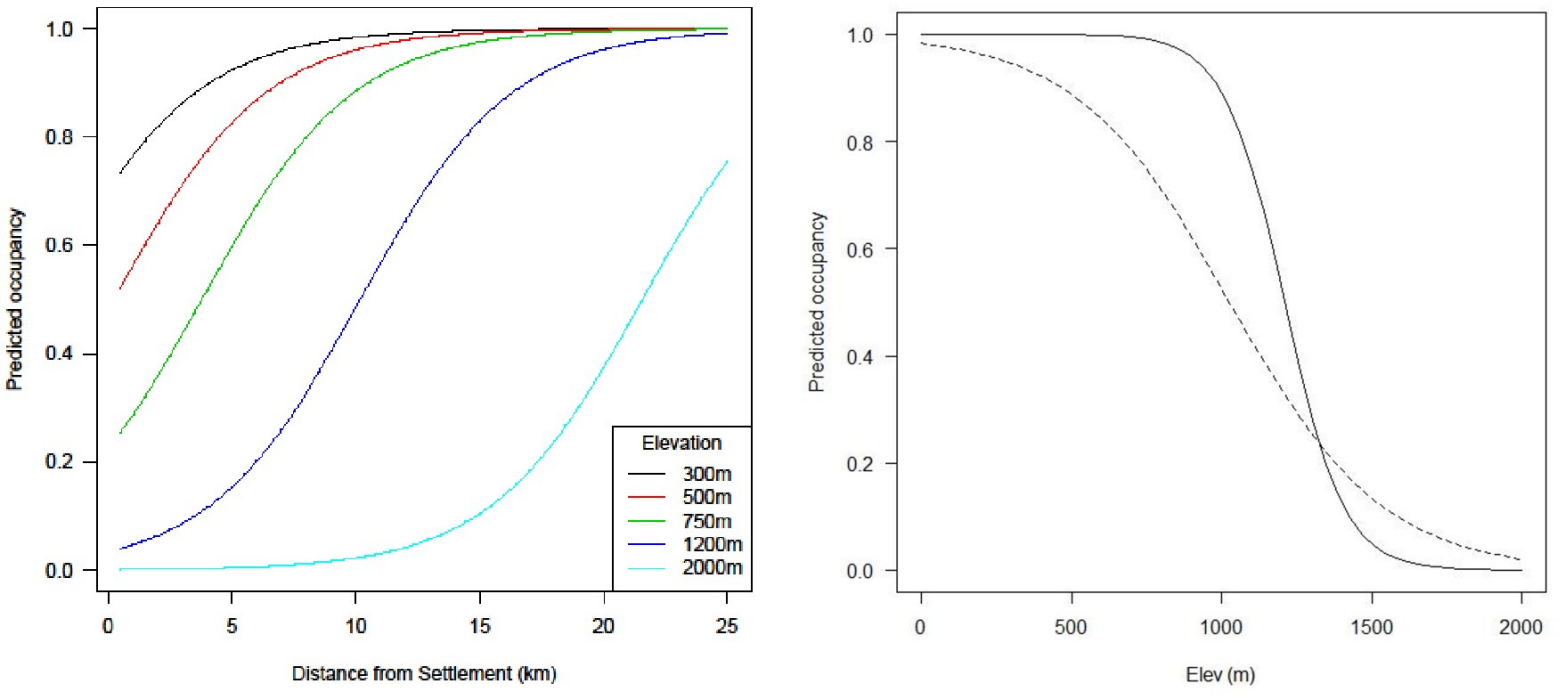
Predicted occupancy for sambar according to elevation and distance from settlement (left) and then for muntjac (solid line) and gaur (dashed line) according to elevation (right) based on the untransformed parameter estimates from the top ranked occupancy models in Temengor Forest Reserve and Royal Belum State Park.

There was evidence of over-dispersion in the datasets for gaur (*p* = 0.10, $\hat{c}$ = 1.3) and muntjac (*p* = 0.11, $\hat{c}$ = 1.6) and the QAICc, which accounts for over-dispersion, was used in the subsequent model selection procedure. The models revealed that gaur, muntjac and sambar occupancy was each negatively influenced by elevation, with sambar occupancy also being positively influenced by distance from settlements (Table 1). Gaur and muntjac showed preference for lower elevation sites, where their respective predicted probability of occupancy from 1.0 to 0.5 at 1,000 and 1,200 m asl, but was close to zero for both >1,800 m asl (Fig 2). Sambar preferred sites at lower elevation and further from settlements, where the predicted probability of occupancy decreases from 1.0 to 0.5 at 500 m asl and when <15 km from settlements (Fig 2).

**Table 1:** Ungulate occupancy (*ψ*) models (w_*i*_ >0) and respective percentage of relative summed model weights (%SMW) for occupancy covariates in Temengor Forest Reserve and Royal Belum State Park.

No	Model	ΔQAICc	w_*i*_	K
*Gaur models for occupancy (ψ) with p (Dist)*				
3.1	*ψ* (Elev)	0.00	0.2333	5
3.2	*ψ* (Lg + Elev + Zone)	0.72	0.1628	7
3.3	*ψ* (Elev + Zone)	1.29	0.1224	6
3.4	*ψ* (Village + Elev)	1.51	0.1096	6
3.5	*ψ* (Lg + Elev)	2.14	0.0800	6
3.6	*ψ* (Lg + Village + Elev + Zone)	3.16	0.0480	8
3.7	*ψ* (.)	3.69	0.0369	4
3.8	*ψ* (Lg + Village + Zone)	3.82	0.0345	7
3.9	*ψ* (Lg + Zone)	3.87	0.0337	6
3.10	*ψ* (Village + Zone)	4.13	0.0296	6
3.11	*ψ* (Village + Elev + Zone)	4.14	0.0294	7
3.12	*ψ* (Lg + Village + Elev)	4.19	0.0287	7
3.13	*ψ* (Zone)	4.97	0.0194	5
3.14	*ψ* (Village)	5.51	0.0148	5
3.15	*ψ* (Lg)	5.82	0.0127	5
3.16	*ψ* (Lg + Village)	8.11	0.0040	6
%SMW: Lg = 40.4, Elev = 81.4, Village = 29.9 and Zone = 48.0				
*Sambar models for occupancy (ψ) with p (Zone + Rain + TN)*				
3.17	*ψ* (Village + Elev)	0.00	0.4681	9
3.18	*ψ* (Village + Elev + Zone)	1.85	0.1856	10
3.19	*ψ* (Lg + Village + Elev)	2.13	0.1614	10
3.20	*ψ* (Lg + Village + Elev + Zone)	4.19	0.0576	11
3.21	*ψ* (Elev + Zone)	4.21	0.0570	9
3.22	*ψ* (Lg + Elev + Zone)	6.44	0.0187	10
3.23	*ψ* (Lg + Zone)	7.15	0.0131	9
3.24	*ψ* (Zone)	7.20	0.0128	8
3.25	*ψ* (Village)	8.41	0.0070	8
3.26	*ψ* (Lg + Village + Zone)	8.92	0.0054	10
3.27	*ψ* (Village + Zone)	9.25	0.0046	9
3.28	*ψ* (Lg + Village)	9.30	0.0045	9
3.29	*ψ* (Elev)	11.72	0.0013	8
3.30	*ψ* (.)	11.72	0.0013	7
3.31	*ψ* (Lg + Elev)	12.17	0.0011	9
3.32	*ψ* (Lg)	13.96	0.0004	8
%SMW: Lg = 26.2, Elev = 95.1, Village = 89.4 and Zone = 35.5				
*Muntjac models for occupancy (ψ) with p (Zone + Obs + TN + Dist)*				
3.33	*ψ* (Elev)	0.00	0.3710	13
3.34	*ψ* (Elev + Zone)	1.54	0.1718	14
3.35	*ψ* (Village + Elev)	2.03	0.1345	14
3.36	*ψ* (Lg + Elev)	2.07	0.1318	14
3.37	*ψ* (Village + Elev + Zone)	3.58	0.0619	15
3.38	*ψ* (Lg + Elev + Zone)	3.65	0.0598	15
3.39	*ψ* (Lg + Village + Elev)	4.11	0.0475	15
3.40	*ψ* (Lg + Village + Elev + Zone)	5.69	0.0216	16
%SMW: Lg = 26.1, Elev = 100.0, Village = 26.6 and Zone = 26.1				

Note: ΔAICc or ΔQAICc = Difference in AICc or QAICc values between each model and the model with the lowest AICc. w*i* = AICc model weight, K = Number of parameters within the model. Models for (*ψ*): Village = Nearest distance of a settlement to the centre of a sampling unit, Elev = Mean elevation for a sampling unit, Lg = Logging intensity index for a sampling unit. Zone = Binary study area covariate. Models for (*p*): Zone = Binary study area covariate, Obs = Categorical observer based covariate, Rain = Binary based covariate on whether it rained or not, Dist = Total distance walked within a sampling unit according to each sign survey, TN = Total number of trap-nights for a given sampling unit according to each sampling occasion.

**Fig 3:**
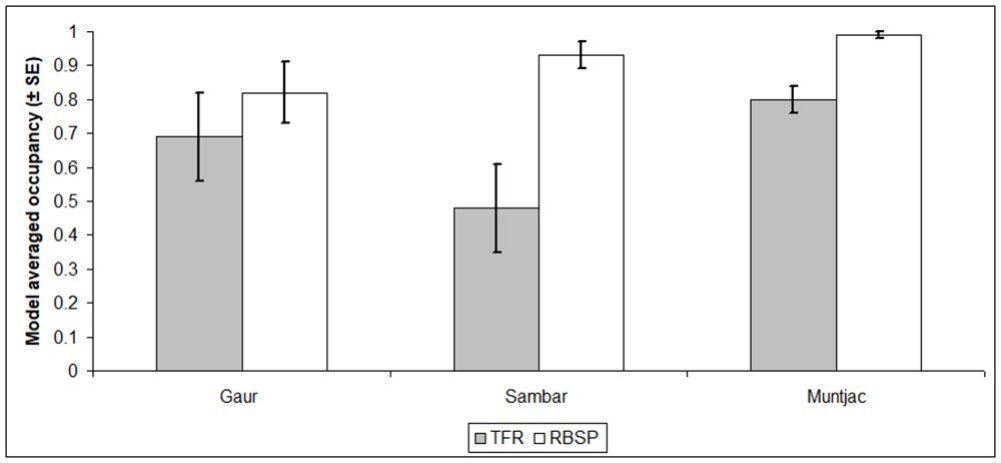
Model averaged occupancy ($\hat{\psi }$) estimates and corresponding standard errors (SE) for three focal ungulate species in Temengor Forest Reserve (grey bars) and Royal Belum State Park (white bars) [note: wild boar is excluded because its occupancy was close to 1.0].

Model averaged occupancy estimates were higher in the primary forests of RBSP compared to the selectively logged forest of TFR for sambar (1.9 times higher) and muntjac (1.2 times; Table 2; Fig 3). Gaur occupancy was higher in RSBP than TFR, but this was non-significant. None of the model averaged occupancy estimates were found to be spatially auto-correlated (Moran’s *I* = 0.01, *P* >0.01).

**Table 2:** Model averaged occupancy ($\hat{\psi }$) and detection probability ($\hat{p}$) estimates for ungulates in Temengor Forest Reserve (TFR) and Royal Belum State Park (RBSP).

Species	Model averaged parameter estimates	TFR	RBSP
Gaur	$\hat{\psi }$ ± SE (95% CIs)	0.69 ± 0.13 (0.44–0.94)	0.82 ± 0.09 (0.64–0.99)
	$\hat{p}$ ± SE for sign surveys	0.53 ± 0.09	0.52 ± 0.08
	$\hat{p}$ ± SE for camera trap surveys	0.09 ± 0.02	0.09 ± 0.02
Sambar ^a^	$\hat{\psi }$ ± SE (95% CIs)	0.48 ± 0.13 (0.23–0.73)	0.93 ± 0.04 (0.85–1.0)
	$\hat{p}$± SE for sign surveys	0.26 ± 0.07	0.72 ± 0.04
	$\hat{p}$± SE for camera trap surveys	0.02 ± 0.01	0.34 ± 0.03
Muntjac ^a^	$\hat{\psi }$ ± SE (95% CIs)	0.80 ± 0.04 (0.72–0.88)	0.99 ± 0.01 (0.97–1.0)
	$\hat{p}$± SE for sign surveys	0.42 ± 0.04	0.48 ± 0.04
	$\hat{p}$± SE for camera trap surveys	0.29 ± 0.04	0.54 ± 0.04

Note: a significant difference between model averaged occupancy estimates of the same species in the different study areas based on Wald test (critical value at α = 0.05).

### Implications for future survey designs

Naive occupancy estimates for wild boar, muntjac, sambar and gaur were consistently higher with the use of indirect sign surveys compared to camera trap surveys, as indicated by a higher number of unique detections (Table 3). Detection probabilities were also consistently higher with the use of three temporal indirect sign survey replicates (of similar effort, 402.6–436.6 km) compared to ten temporal camera trap survey replicates for all species except muntjac (Table 3). The probability of a false absence for all species with either method was not greater than 0.15, with the exception of gaur from camera trap surveys (Table 3). The results collectively showed that for the four target species and with the use of the current field design, indirect sign surveys performed better than camera trapping in terms of detection efficiency and correspondingly produced higher occupancy estimates (Table 3). Based on the constant model *ψ*(.)*p*(.) and estimates from sign surveys (Table 3), a predicted total number of 29 (4 km^2^), 69 (1 km^2^), 107 (4 km^2^) and 93 (16 km^2^) sites would be required to be surveyed for wild boar, muntjac, sambar and gaur, respectively, in order to obtain precise estimates of occupancy with a standard error of 0.05.

**Table 3:** Occupancy ($\hat{\psi }$) and detection probability ($\hat{p}$) estimates with corresponding standard errors (SE) from the constant model *ψ*(.)*p*(.) and probability of a false absence with the use of sign surveys and camera trapping for ungulates from all combined sites in Temengor Forest Reserve and Royal Belum State Park.

Species	Sign surveys			Camera trapping		
	$\hat{\psi }$± SE	$\hat{p}$ ± SE	Probability of a false absence	$\hat{\psi }$ ± SE	$\hat{p}$ ± SE	Probability of a false absence
Wild boar	0.99 ± 0.01	0.80 ± 0.02	0.008	0.95 ± 0.02	0.45 ± 0.02	0.003
Muntjac	0.85 ± 0.03	0.47 ± 0.01	0.149	0.84 ± 0.02	0.69 ± 0.01	0.000
Sambar	0.66 ± 0.05	0.64 ± 0.03	0.047	0.43 ± 0.04	0.28 ± 0.02	0.037
Gaur	0.74 ± 0.09	0.55 ± 0.06	0.091	0.50 ± 0.13	0.09 ± 0.02	0.389

## Discussion

The combined sign and camera trap sampling approach used in our study sheds new and important light on Malaysia’s under threat ungulates. It provides the first set of robust occupancy estimates from Permanent Reserved Forest that is primarily classified as Production Forests and highlights the importance of this forest type in supporting populations of threatened wildlife, which in this case supports a guild of threatened large carnivores [16,34]. However, the results further underscore the importance of protected areas, for which the primary and relatively better protected forests of RBSP had higher ungulate occupancies. The results stress the need to protect forest below 1,000 m asl because it represents vital habitat that is subjected to logging and at risk of conversion to monoculture plantations that offer few habitat benefits for the threatened ungulates in our study [14].

Considering each of the target ungulate species individually, the widespread (near 100% occupancy) finding for wild boar was unsurprising because as a non-ruminant omnivore it is a habitat generalist [20,35]. However, it is often hunted for meat and trapped as a crop pest and this result is therefore reassuring when considering the dramatic decline of Peninsula of Malaysia’s other wild pig species, the bearded pig (*Sus barbatus*) [36]. Bearded pig, a non-ruminant species, is also hunted but differs from wild boar ecology; they are migratory and follow mast fruiting events across large swatches of forest that makes them more susceptible to forest fragmentation and the loss of fruiting trees to selective logging [10].

For muntjac, occupancy decreased as elevation increased (particularly >1,800 m asl), but with a strong preference for lowland-hill (0–750 m asl) dipterocarp forest, where occupancy was 0.99, as also found in a muntjac habitat suitability study from India [37]. Sambar occupancy also decreased with increasing elevation in combination with closer proximity to settlements (*ψ* = 1.0 at 500 m asl when > 15 km from settlements and *ψ* < 0.1 at 1,200 m asl when < 4 km), a similar finding to a study from Thailand where sambar sign was two-fold higher in lowland forest than montane forest [38].

The influence of elevation is in part explained by its association with biophysical parameters such as temperature and precipitation that change with altitude to shape the local diversity of plants and habitat structure, which influence mammal density, distribution and diversity [39,40]. For example, at higher elevations substantial declines have been documented in: fleshy fruit availability, including declines in fig (*Ficus* sp.) abundance—a key food item of ruminant frugivorous species in rainforest >700 m asl; and, the quality of foliage and browse materials [8,41,42].

For sambar, an additional explanatory factor was identified, with lower occupancies recorded closer to human habitation, which follows similar patterns from India and Indonesia [22,37]. The link between commercial logging and bushmeat hunting has been demonstrated throughout the tropics [6,11]. Corroborating our finding of proximity to human habitation and its link to higher hunting pressure and therefore lower occupancy is the supporting information obtained from interview surveys with 190 Orang Asli respondents [18]. From this survey, some (12.7%) respondents in TFR said that loggers hunted bushmeat and a higher number (18.5%) of respondents from TFR, as opposed to those from RBSP (3.1%), disclosed that they had been approached by local people to assist their hunting, primarily of muntjac, sambar, gaur, pangolins and turtles [18].

For gaur, the predicted probability of occupancy reduced from 0.5 at 1,000 m asl to 0.0 at 1,800 m asl, indicating an altitudinal range limit for the species and a general preference to occupy lowland-hill dipterocarp forest. From three radio-tracked gaur in the Ulu Lepar Forest of Pahang state, Peninsular Malaysia, habitat below 61 m asl was found to be intensively used in a study area that ranged up to 1079 m asl [43]. In the Tenasserim–Dawna Mountains of Thailand, gaur signs were more abundant in lowland than hill forest [38] and in India, low-lying areas were preferred gaur habitat [44]. Grass and bamboo are favoured food items for gaur, while mineral licks, which are more abundant in lowland areas, provide them with important dietary supplements [38]. We found no discernible difference for gaur between logged and unlogged areas that, as a grazing ruminant like banteng (*Bos javanicus*), may benefit from selective logging practices that result in grassy clearings and bamboo patches [10]. Further, from the 35 natural mineral licks recorded in our study areas, none were >700 m asl (D.M. Rayan unpublished data).

The combined use of camera trap and indirect sign survey data increased the detection of multiple prey species. The sign surveys outperformed the camera trap surveys in obtaining a greater number of unique detections for each of the focal species, and consequently yielded higher detection probabilities. This finding is important because low detection probabilities make it difficult for the occupancy models to distinguish reliably between sites with a low species detection probability (i.e. <0.15) and sites where a species is truly absent [31]. Taking gaur as an example, the use of camera trap data alone may not have been sufficient to enable inferences to be confidently made on its occupancy status and associated habitat correlates. This detection failure is important because our study responds to a call by the Malaysian government to monitor tiger ungulate prey occupancy in one of its three identified priority sites, in our case RBSP [45].

The issue of scale in the interpretation of the effects of home range size and the use of arbitrary grid cells for occupancy needs careful consideration as it serves an important criterion to satisfy the corresponding assumptions. A basic assumption of the occupancy estimation method is that sites are closed to changes in occupancy during the repeat surveys [31] and given that none of the focal species are migratory, it was assumed that the population was demographically closed. However, since the sampling design contained 4 km^2^ grid cells that were next to each other and may not be proportionate to the home range size of the species, this might lead occupancy to being interpreted as ‘intensity of use’ [32]. To reduce the chance of the species moving between sites within each sampling period and to maintain the state variable as occupancy, grid cells of 4 km^2^ were reduced or combined to be larger than the largest home range sizes for each species. The definition of a larger arbitrary sampling unit size than the approximate home range values for gaur, sambar, wild boar and muntjac, is an additional criterion to satisfy the closure assumption at the species level, as suggested by MacKenzie et al. (2006) [31].

Another important issue that influences the choice of survey method is cost. Assuming that the costs for basic field equipment (transportation, camping equipment, etc.) are equal for both survey methods, camera trapping would incur an additional cost of US$42,000, as the 70 camera traps under the current design cost US$600 each. Even if camera trapping were to reduce the time spent in the field as opposed to conducting sign surveys, the overall costs are still expected to be much higher due to the high initial equipment cost of camera traps. Additionally, if camera trap damage and theft or malfunction is high, this will inevitably result in a reduced data set and incur additional costs for replacement camera units or for repairing damaged cameras, which may not necessarily be cost efficient for a long-term approach. Sign surveys would be less expensive to conduct under the current survey design, but unlike camera traps would only record data on the target species.

Prior to our study, ungulate occupancy surveys were conducted in Endau-Rompin National Park, which straddles Johor and Pahang states [46], and Sungai Yu Forest Reserve in Pahang state [47]. Notably, these and other surveys identified a contraction in the distributions and declines in the relative abundances of sambar and muntjac, as inferred through photo trapping rates from a nationwide survey [48]. Based on this, since 2009, the Malaysian Department of Wildlife and National Parks issued a six-year hunting moratorium on sambar and muntjac, while assigning the highest protection status (Totally Protected Species) to bearded pig. The moratorium has since been extended by another six years until 2021. Gaur is a fully protected species under Malaysian law. The hunting and trafficking of this species carries a mandatory fine of no less than one hundred thousand Ringgit and up to five hundred thousand Ringgit (approx. US$25,000–125,000), and imprisonment for up to five years for its poaching. While laws appear to be adequate, corresponding wildlife enforcement and specialised anti-poaching patrols particularly in these Permanent Reserved Forests continues to be poor or non-existent [7,34].

Reconciling the need for economic development and biodiversity conservation is a global challenge [49]. Our study affirms the primary importance of protected areas for managing threatened wildlife in Peninsular Malaysia. However, for the selectively logged forest it also stressed its equal importance as a refuge for threatened species, especially when connected to a protected area. However, the clearance of selectively logged over forest for timber latex clone rubber plantations [14] or any other monoculture plantation is a growing conservation concern because its constitutes a major habitat loss for ungulate populations, increases access for poachers and threatens apex predators, such as the tiger [34]. The options to manage mosaic forests that offer simultaneous benefits to wildlife, rural communities and economic growth, include enhanced silvicultural practices. For example, with reduced-impact logging and forest management protocols that reduce timber extraction intensity, as well as payment for ecosystem services linked to watershed forest protection. This change will require embarking on a different development trajectory that avoids converting logged-over forests into monoculture plantations [50]. An approach that will also require substantial political will to assure that good forestry practice and governance that includes wildlife protection from poaching is administered to manage the Peninsular’s three million hectares of remaining production forest.

## Supporting information

S1 Table Number of sampling units, detections and naive occupancy from combined camera trap and sign survey data for ungulates in Temengor Forest Reserve (TFR) and Royal Belum State Park (RBSP). (DOCX)

S2 Table Ungulate detection probability (p) models (wi >0), with ψ (Lg + Set + Elev + Zone) and respective percentage of relative summed model weights (%SMW) for detection probability covariates in Temengor Forest Reserve and Royal Belum State Park. Note: ΔAICc = Difference in AICc values between each model and the model with the lowest AICc. w*i* = AICc model weight, K = Number of parameters within the model. Models for (*ψ*): Set = Nearest distance of a settlement to the centre of a sampling unit, Elev = Mean elevation for a sampling unit, Lg = Logging intensity index for a sampling unit. Zone = Binary study area covariate. Models for (*p*): Zone = Binary study area covariate, Obs = Categorical observer based covariate, Rain = Binary based covariate on whether it rained or not, Dist = Total distance walked within a sampling unit according to each sign survey, TN = Total number of trap-nights for a given sampling unit according to each sampling occasion.
(DOCX)
