# Supplementary material for: Managing threatened ungulates in logged-primary forest mosaics in Malaysia
Source: PLoS One. 2020 Dec 14;15(12):e0243932. doi: 10.1371/journal.pone.0243932 (PMC7735589; doi:10.1371/journal.pone.0243932)
Supplement: S1 Table — (DOCX) [file pone.0243932.s001.docx]

**S1 Table.** **Number of sampling units, detections and naive occupancy from combined camera trap and sign survey data for ungulates in Temengor Forest Reserve (TFR) and Royal Belum State Park (RBSP)**

| **Species** | **Parameters** | **TFR** | **RBSP** |
| --- | --- | --- | --- |
| Gaur | Number of sampling units (16 km^2^ grid cells) | 22 | 20 |
|  | Number of sampling units with detections | 13 | 15 |
|  | Naive occupancy (%) | 59 | 75 |
| Sambar | Number of sampling units (4 km^2^ grid cells) | 70 | 70 |
|  | Number of sampling units with detections | 21 | 65 |
|  | Naive occupancy (%) | 30 | 93 |
| Wild pig | Number of sampling units (4 km^2^ grid cells) | 70 | 70 |
|  | Number of sampling units with detections | 67 | 70 |
|  | Naive occupancy (%) | 96 | 100 |
| Muntjac | Number of sampling units (1 km^2^ grid cells) | 280 | 280 |
|  | Number of sampling units with detections | 173 | 237 |
|  | Naive occupancy (%) | 62 | 85 |
