# Supplementary material for: Managing threatened ungulates in logged-primary forest mosaics in Malaysia
Source: PLoS One. 2020 Dec 14;15(12):e0243932. doi: 10.1371/journal.pone.0243932 (PMC7735589; doi:10.1371/journal.pone.0243932)
Supplement: S2 Table — Note: ΔAICc = Difference in AICc values between each model and the model with the lowest AICc. wi = AICc model weight, K = Number of parameters within the model. Models for (ψ): Set = Nearest distance of a settlement to the centre of a sampling unit, Elev = Mean elevation for a sampling unit, Lg = Logging intensity index for a sampling unit. Zone = Binary study area covariate. Models for (p): Zone = Binary study area covariate, Obs = Categorical observer based covariate, Rain = Binary based covariate on whether it rained or not, Dist = Total distance walked within a sampling unit according to each sign survey, TN = Total number of trap-nights for a given sampling unit according to each sampling occasion. (DOCX) [file pone.0243932.s002.docx]

**S2 Table.** **Ungulate detection probability (*p*) models (w*_i_* >0), with *ψ* (Lg + Set + Elev + Zone) and respective percentage of relative summed model weights (%SMW) for detection probability covariates in Temengor Forest Reserve and Royal Belum State Park**

| **No** | **Candidate Models for detection probability with a global (ψ) model** | **ΔAICc** | **w*_i_*** | **K** |
| --- | --- | --- | --- | --- |
| *Gaur* | | | | |
| 2.1 | *p* (Dist) | 0.00 | 0.3295 | 8 |
| 2.2 | *p* (Zone + Dist) | 0.12 | 0.3104 | 10 |
| 2.3 | *p* (TN +Dist) | 1.73 | 0.1388 | 9 |
| 2.4 | *p* (Zone +TN +Dist) | 2.32 | 0.1033 | 11 |
| 2.5 | *p* (.) | 3.26 | 0.0646 | 7 |
| 2.6 | *p* (TN) | 4.80 | 0.0299 | 8 |
| 2.7 | *p* (Zone) | 6.35 | 0.0138 | 9 |
| 2.8 | *p* (Zone +TN) | 7.03 | 0.0098 | 10 |
| %SMW: Zone = 43.7; TN = 28.2 and Dist = 88.2 | | | | |
| *Sambar* | | | | |
| 2.11 | *p* (Zone + Rain + TN) | 0.00 | 0.6021 | 11 |
| 2.12 | *p* (Zone + Rain + TN + Dist) | 1.55 | 0.2774 | 12 |
| 2.13 | *p* (Zone + TN) | 5.45 | 0.0395 | 10 |
| 2.14 | *p* (Zone + Obs + Rain + TN) | 5.59 | 0.0368 | 16 |
| 2.15 | *p* (Zone + TN + Dist) | 6.69 | 0.0212 | 11 |
| 2.16 | *p* (Zone + Obs + Rain + TN + Dist) | 7.43 | 0.0147 | 17 |
| 2.17 | *p* (Zone + Obs + TN) | 9.33 | 0.0057 | 15 |
| 2.18 | *p* (Zone + Obs + TN + Dist) | 10.85 | 0.0027 | 16 |
| %SMW: Zone = 100.0; Rain = 93.1; TN = 100.0 and Dist = 31.6 | | | | |
| *Muntjac* | | | | |
| 2.19 | *p* (Zone + Obs + TN + Dist) | 0.00 | 0.7436 | 16 |
| 2.20 | *p* (Zone + Obs + Rain + TN + Dist) | 2.13 | 0.2564 | 17 |
| %SMW: Zone = 100.0; Obs =100.0 Rain = 26.0 ; TN = 100.0 and Dist = 100.0 | | | | |

Note: ΔAICc = Difference in AICc values between each model and the model with the lowest AICc. w*i* = AICc model weight, K = Number of parameters within the model. Models for (*ψ*): Set = Nearest distance of a settlement to the centre of a sampling unit, Elev = Mean elevation for a sampling unit, Lg = Logging intensity index for a sampling unit. Zone = Binary study area covariate. Models for (*p*): Zone = Binary study area covariate, Obs = Categorical observer based covariate, Rain = Binary based covariate on whether it rained or not, Dist = Total distance walked within a sampling unit according to each sign survey, TN = Total number of trap-nights for a given sampling unit according to each sampling occasion.
